# System-level factors influencing refugee women's access and utilization of sexual and reproductive health services: A qualitative study of providers’ perspectives

**DOI:** 10.3389/fgwh.2022.1048700

**Published:** 2022-12-14

**Authors:** Milkie Vu, Ghenet Besera, Danny Ta, Cam Escoffery, Namratha R. Kandula, Yotin Srivanjarean, Amanda J. Burks, Danielle Dimacali, Pabitra Rizal, Puspa Alay, Cho Htun, Kelli S. Hall

**Affiliations:** ^1^ Department of Preventive Medicine, Feinberg School of Medicine, Northwestern University, Chicago, IL, United States; ^2^ Department of Behavioral, Social, and Health Education Sciences, Rollins School of Public Health, Emory University, Atlanta, GA, United States; ^3^ Nell Hodgson Woodruff School of Nursing, Emory University, Atlanta, GA, United States; ^4^ Department of Medicine, Feinberg School of Medicine, Northwestern University, Chicago, IL, United States; ^5^ Center for Pan Asian Community Services, Atlanta, GA, United States; ^6^ Emory University Physician Assistant Program, School of Medicine, Emory University, Atlanta, GA, United States; ^7^ Heilbrunn Department of Population & Family Health, Mailman School of Public Health, Columbia University, New York, NY, United States

**Keywords:** refugee women, healthcare providers, transportation services, interpretation services, patient navigators, sexual and reproduction health, healthcare system, implementation science

## Abstract

Refugee women have poor outcomes and low utilization of sexual and reproductive health (SRH) services, which may be driven by access to and quality of SRH services at their resettled destinations. While healthcare providers offer valuable insights into these topics, little research has explored United States (U.S.) providers' experiences. To fill this literature gap, we investigate U.S. providers' perspectives of healthcare system-related factors influencing refugee women's access and utilization of SRH services. Between July and December 2019, we conducted in-depth, semi-structured interviews with 17 providers serving refugee women in metropolitan Atlanta in the state of Georgia (United States). We used convenience and snowball sampling for recruitment. We inquired about system-related resources, facilitators, and barriers influencing SRH services access and utilization. Two coders analyzed the data using a qualitative thematic approach. We found that transportation availability was crucial to refugee women's SRH services access. Providers noted a tension between refugee women's preferred usage of informal interpretation assistance (e.g., family and friends) and healthcare providers’ desire for more formal interpretation services. Providers reported a lack of funding and human resources to offer comprehensive SRH services as well as several challenges with using a referral system for women to get SRH care in other systems. Culturally and linguistically-concordant patient navigators were successful at helping refugee women navigate the healthcare system and addressing language barriers. We discussed implications for future research and practice to improve refugee women's SRH care access and utilization. In particular, our findings underscore multilevel constraints of clinics providing SRH care to refugee women and highlight the importance of transportation services and acceptable interpretation services. While understudied, the use of patient navigators holds potential for increasing refugee women's SRH care access and utilization. Patient navigation can both effectively address language-related challenges for refugee women and help them navigate the healthcare system for SRH. Future research should explore organizational and external factors that can facilitate or hinder the implementation of patient navigators for refugee women's SRH care.

## Introduction

Since the passage of the Refugee Act of 1980, the United States (U.S.) has admitted more than 3.1 million refugees (1). While the admission number has fluctuated each year based on global events and the priorities of different administrations, around half of resettled refugees are female (2, 3). Refugee women often face challenges and risks that are distinct from those encountered by their male counterparts (4). In particular, their history of pre-migration experiences, including torture, exposure to war, gender-based violence, and post-traumatic stress disorder (5, 6), as well as their post-migration living difficulties (7) can have negative implications for their sexual and reproductive health (SRH) (8). Indeed, U.S. and/or globally resettled refugee women are shown to have poor SRH outcomes and low knowledge and utilization of SRH services (e.g., contraceptives, cervical cancer screening, or HPV vaccination) (9–26).

Besides refugee women's pre-migration history and post-migration living conditions, a key but understudied determinant of SRH disparities experienced in this population may be access to, as well as the quality and appropriateness of SRH services at their resettled destinations (27–29). According to Liu and colleagues' framework (30), access means “an individual's ability to position oneself to receive healthcare services,” while utilization “presumes access and requires effective information exchange during a healthcare encounter.” Both healthcare access and utilization are influenced by the structure and operation of the system in which individuals receive care (30).

A recent systematic review synthesized evidence from 28 studies on access to preventive SRH care for refugee, asylum seeker, and internally displaced women in high-, middle-, and low-income host countries (31). The review found that while most existing studies have explored barriers of access, not many have focused on enablers of access. Among many challenges, in high-income host countries, refugee women face barriers in navigating and understanding the health system and making appointments for SRH care. Critically, the authors discussed how relatively few of the reviewed studies have investigated healthcare providers' perspectives and experiences providing care to refugee women.

The perspectives of healthcare providers on these issues are important as providers are at the forefront of caring for SRH needs in this population and can offer valuable insights into clinical encounters and possible challenges (32). Additionally, providers can speak about not only organizational barriers but also organizational resources and facilitators related to the delivery of SRH care. An understanding of these healthcare system-level factors is critical to ensuring health equity, as it allows for the allocation of resources to meet particular needs (33), which can subsequently increase refugee women's SRH access and utilization. Moreover, from an implementation science perspective, providers offer crucial insights on organizational inner context factors (e.g., existing programs, staffing, resources, funding) and outer context factors (e.g., interorganizational environment and networks, patient characteristics and needs) that can inform future implementation efforts to increase SRH access and care for refugee women (34).

Existing studies with providers serving refugee women have been predominantly conducted in non-U.S. settings such as Australia, Canada, or European countries (29, 35–44), with few studies based in the U.S. (32, 45–48). The non-U.S. literature has uncovered several challenges refugee women encounter in accessing services, as well as obstacles that clinicians faced in delivering high-quality, culturally-relevant health services for refugee women (29, 35, 37–41). Yet, the generalizability of non-U.S. findings to the U.S. context may be limited given differences in health insurance policy and structure of the healthcare systems. Moreover, some of the few U.S.-based studies do not distinguish between providers serving refugees, immigrants, or foreign-born individuals (32, 47), despite the fact that people's circumstances for migration as well as their official migration status greatly matter for healthcare (49–51).

To fill these gaps in the literature, we conducted a qualitative study with U.S. healthcare providers in metropolitan Atlanta about their experiences serving refugee women's SRH needs. Specifically, our study explored providers' perspectives of healthcare system-related resources, facilitators, and barriers influencing refugee women's access and utilization of SRH services. Our study uses a phenomenological approach, which investigates several individuals' shared experiences of a concept or phenomenon (e.g., providers’ experiences with refugee women's SRH access and utilization) (52).

## Materials and methods

### Study setting and participants

Our study setting was the metropolitan Atlanta area, located within the state of Georgia. Georgia has historically been among the U.S. states that receive the highest numbers of resettled refugees (2, 3). For example, between 2010 and 2020, Georgia was the initial resettled destination of 4% of all refugees in the U.S. (53). A large number of refugees are concentrated in the metropolitan Atlanta area (54–57). In particular, in the past three decades, the city of Clarkston in the metropolitan Atlanta area has served as the primarily hub for refugee resettlement in Georgia and has resettled more than 40,000 refugees (58). The top countries of origins for refugees in Georgia are Burma, Congo, Syria, Afghanistan, and Bhutan (59); these are also the top countries of origins for refugees resettled in the U.S. (53).

The numbers of refugees resettled in Georgia in the years preceding the conduct of this study decreased considerably due to the reduction in the cap on refugee admissions set by the Trump Administration. For example, while 3,017 refugees were initially resettled in Georgia in fiscal year 2016 (2), only 1,182 were resettled in fiscal year 2019 (3). Moreover, despite being one of the top states in terms of refugee resettlements, the state of Georgia reportedly spends little to no state dollars to fund programs meant to specifically help refugees (60, 61). At the time of our study, Georgia ranked third worst in the U.S. regarding the uninsured rate (62), and it had not adopted a full expansion of Medicaid for its resident (63).

Between July and December 2019, we recruited 26 refugee women and 17 healthcare providers to participate in our study. The data analyzed in this manuscript focused on the 17 healthcare providers. Eligibility criteria for providers included (1) identifying as a healthcare provider and (2) previously or currently providing SRH services (e.g., family planning, sexually transmitted diseases testing, cervical cancer screening, HPV vaccination, or perinatal care) to refugee women in the metropolitan Atlanta area. We sampled providers from different occupations (e.g., physicians, nurse practitioners, registered nurses, other clinic staff). Providers were first identified through word-of-mouth recruitment at clinics that refugee women had indicated, in previous interviews, that they attended for SRH services. Additionally, we contacted community-based organizations serving refugee populations for names of clinics or providers that regularly saw refugee women for SRH visits. We also utilized snowball sampling and asked providers who were interviewed to identify other eligible providers. Providers worked at several different clinics that cared for refugee women, including federally qualified health centers (FQHCs) and charitable, volunteer-operated clinics. The *a priori* sample size of 15–20 was selected based on past qualitative research of similar topics (32, 39, 43, 45, 47, 48) and recommendations for sample size needed to achieve code and meaning saturation (64). The Emory University Institutional Review Board approved this study (IRB00142813).

### Data collection

Prior to the start of each interview, each provider gave verbal consent and answered a brief survey about their sociodemographic information. Each interview lasted 30–60 min. Participants were compensated with a $20 gift card upon interview completion. All interviews were conducted in person by two authors (MV and GB). Both interviewers were doctoral candidates trained in behavioral and social sciences and in public health, with prior formal coursework and professional experience in qualitative research. Both were also women of color and first- or second-generation immigrants in the U.S. They both had extensive personal networks, as well as professional research experience with migrants and refugees in the U.S.

**Figure 1:**
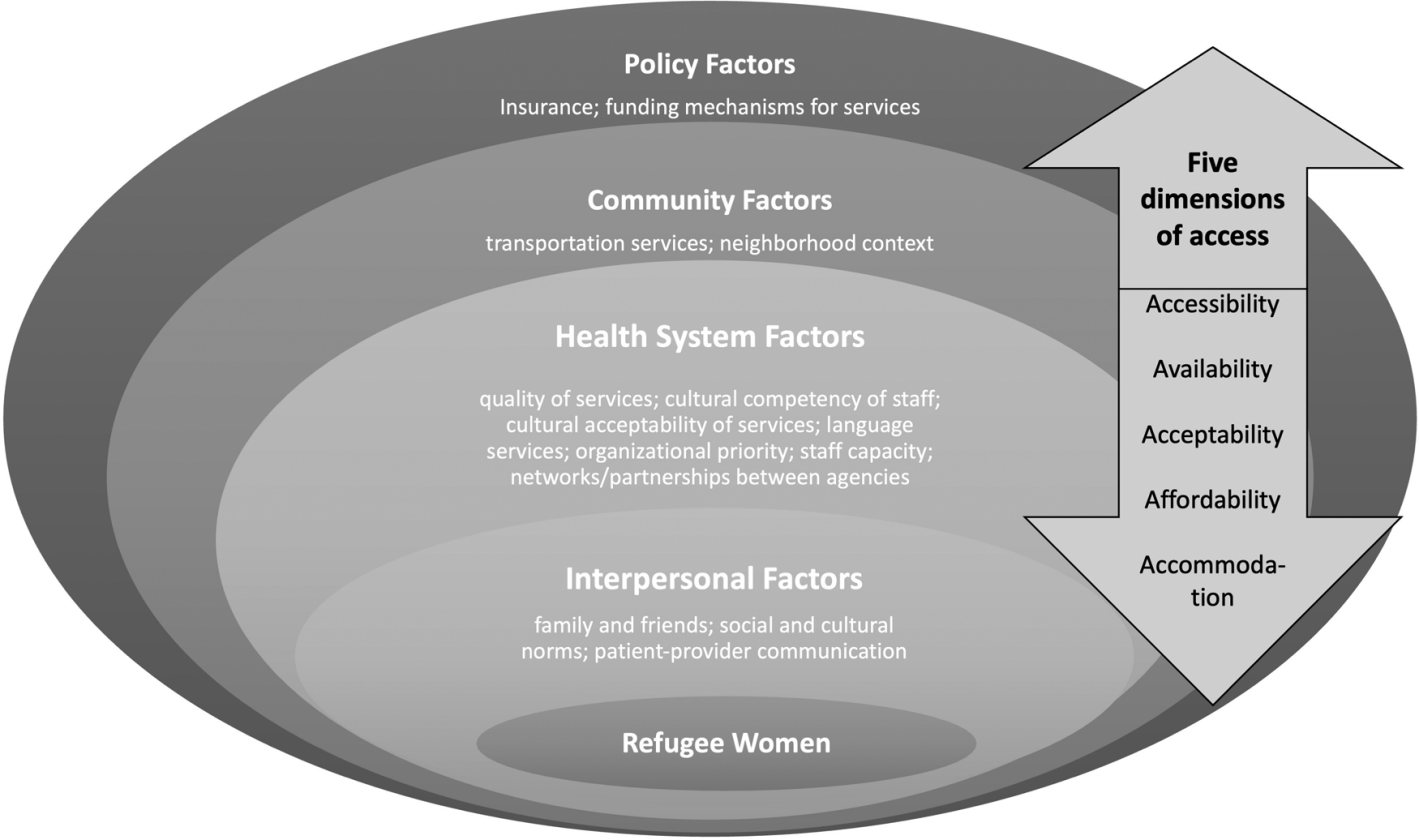
Conceptual model of the study.

The semi-structured interview guide was developed based on a conceptual model (Figure 1) that integrates the Socioecological Framework (65) and Penchansky and Thomas' Theory of Access (66). The Sociological Framework describes how engagement in SRH services utilization can be influenced by factors at multiple levels (e.g., interpersonal, healthcare systems, community, and policy) (37, 65). Interview questions also assessed the five dimensions of access (66) which involve accessibility (i.e., services proximity to ethnic enclaves or communities), availability (i.e., sufficient resources, including language services, to meet SRH needs), acceptability (i.e., services are culturally acceptable to patients), affordability (for both service providers and community members), and accommodation (defined as relationships between the structure/resources of the organization and the patient's ability to accommodate these factors, i.e., refugee women's ability to follow through on referral to other care settings).

### Data analysis

All interviews were audio-recorded and transcribed verbatim by a professional audio transcription company. We analyzed the data using an inductive qualitative thematic analysis approach (67). While the theory-driven research questions outlined by the research team provided the domains of relevance for the analysis (68), identified themes and patterns were strongly data-driven. In other words, our findings arose primarily and directly from the analysis of the raw qualitative data and not *a priori* models or a pre-existing coding frame (68, 69). We established trustworthiness during each phase of thematic analysis by: (1) familiarizing ourselves with the data; (2) generating initial inductive codes from the qualitative data; (3) searching for themes; (4) reviewing themes; (5) defining and naming themes; and (6) producing the report (67). First, two researchers (MV and GB) read five transcripts carefully and developed a codebook with definitions (based on the initial inductive codes), inclusion and exclusion criteria, and examples. The two researchers then independently coded the initial five transcripts using the codebook. Subsequently, the two researchers met to compare coding results and reconciled any discrepancies through discussion. The two researchers then each coded six transcripts of the remaining twelve transcripts using the codebook. A third researcher (DT) reviewed these twelve coded transcripts. Any discrepancies were reconciled through discussion among the three researchers. We used researcher triangulation, vetted themes and subthemes by research team members, and reached consensus on themes (67).

To establish the significance of patterns and meaning while balancing the controversy regarding whether to quantify qualitative results (70), we operationalized the frequency of themes that appeared in interviews as “all” (100% of interviews), “almost all” (90%–99%), “most” (70%–89%), “the majority” (50%–69%), “several” (20%–49%), and “a few” (less than 20%) (71). We employed several techniques to establish and enhance validity, rigor, and trustworthiness in qualitative research, including articulating data collection and analysis decisions, providing verbatim transcription, exploring rival explanations, performing a literature review, peer debriefing, negative case analysis, and close collaboration with community partners (72–74). We used MAXQDA 2020 (75) for all qualitative data analyses and data management activities. Descriptive statistics were generated for sociodemographic characteristics in Stata 16 (76).

## Results

### Participant characteristics

Table 1 displays providers' sociodemographic characteristics. The majority of providers were female (88.2%). Approximately a half identified as White and a third identified as Asian. On average, providers had 14.2 and 6.5 years of experience in healthcare and in working with refugee women, respectively. The most common occupations were physicians (29.4%) and registered nurses (29.4%). Providers mentioned that the majority of refugee patients they saw were from South and Southeast Asia (e.g., Bhutan, Nepal, and Burma), the Middle East (e.g., Syria, Iraq, and Afghanistan), and East and Central Africa (e.g., Eritrea, Somalia, and the Democratic Republic of Congo).

**Table 1:** Sociodemographic characteristics of providers.

Variable	Total
	*N* = 17
	*N* (%) or M (SD)
Sex	
Male	2 (11.8%)
Female	15 (88.2%)
Race	
White	9 (52.9%)
Asian	6 (35.3%)
Native Hawaiian or Other Pacific Islander	1 (5.9%)
Other	1 (5.9%)
Country of origin	
United States	10 (58.8%)
Elsewhere	7 (41.2%)
Years of experience in healthcare	14.2 (9.8)
Years of experience working with refugee women	6.5 (8.3)
Occupation	
Physicians	5 (29.4%)
Nurse practitioners	4 (23.5%)
Registered nurses	5 (29.4%)
Other	3 (17.6%)

### Healthcare system-related factors influencing refugee women's access and utilization of SRH services

Providers described a range of healthcare system-related factors influencing refugee women's access and utilization of SRH services: clinic location and transportation availability; differences in perceived acceptability of interpretation services; limited clinical-level capability to provide SRH services; and challenges with current referral systems for SRH services. These key emergent themes are further described below and summarized in Table 2.

**Table 2:** Healthcare system-related resources, opportunities, and barriers influencing refugee women's access and utilization of sexual and reproductive health services.

Clinic location and transportation availability were key factors impacting access to sexual and reproductive health services
Differences in perceived acceptability of interpretation services: • Refugee women preferred using family members or friends as informal interpreters to telephone services such as the Language Line • Providers, however, raised issues with using informal interpreters, particularly with privacy concerns and uncertain quality of translation • In-person interpreters provided by the clinic or linguistically-concordant patient navigators could successfully resolve language barriers for women
Limited clinic-level capability to provide sexual and reproductive health services to refugee women:
• Limitations on patients that can be seen based on insurance policy • Limited human resources or time • Lack of funding for and inadequate offering of services
Challenges with current referral system for sexual and reproductive health services:
• Providers frequently referred female refugee patients to other settings (e.g., public hospitals, other FQHCs, or non-profit organizations) for services that were not provided at their particular clinics (e.g., prenatal care, HPV vaccination, and SRH diagnostic tests) • Barriers impeding refugee women's follow-through on referrals included restrictions based on catchment areas, logistical difficulties, and issues with language or transportation • Patient navigators were helpful for ascertaining refugee women's follow-through on referrals

#### Impact of location and transportation on access to sexual and reproductive health services

Providers often described how clinics that were centrally located within refugee communities facilitated access to services. For instance, a registered nurse said: “The location of the clinic is … very centrally located  … A lot of women can walk to clinic” (#17). At the same time, several providers mentioned that for refugee women who lived in other parts of metropolitan Atlanta or for clinics that were less reachable on foot, transportation difficulties could be a major access barrier. A registered nurse discussed: “[Refugee women] are often dependent on family members, community members or individuals at the place of worship to bring them to clinic … they're not really in charge of their own time” (#16). To overcome this issue, one clinic offered their patients free transportation services to the clinic. A program coordinator described: “A lot of refugee women … don’t have transportation, but we have a transportation team on site … we do provide the transportation service” (#03).

#### Provision of interpretation services and differences in perceived acceptability of services

Several providers stated that limited English proficiency was a major barrier for services access and utilization. Most providers discussed the use of on-demand telephone interpretation services. However, several providers mentioned that women with limited English proficiency would often prefer having family members or friends serve as interpreters to using telephone services. A physician said: “We typically try to use the Language Line if it's at all possible, just because that's a more accurate way of doing it … [Patients] typically will decline the phone and prefer to use their friend or family member” (#08).

Several providers discussed certain disadvantages of using on-demand telephone interpretation services. A midwife explained: “Midwifery care tends to be a conversation and we would sit in the room with somebody and provide labor support. A lot of the communication's nonverbal … The Language Line doesn’t work for casual conversation. The focus of using the Language Line is very clinical. It's not cultural … A challenge is the cost of the Language Line and getting the right translation services … They need advanced notice … before their appointment in order to get the right language available” (#09).

While having refugee women's family members or friends serving as interpreters could resolve some challenges with using telephone interpretation services, providers had mixed perceptions about this practice. A nurse practitioner (#05) remarked: “Especially when you’re talking about sexual and reproductive health, having family members is always going to be a challenge in … feeling confident that the answers that they’re giving are accurate … When we’re talking about vaginal discharge, that can be an uncomfortable conversation that you wouldn’t want to have in front of your neighbor.” Another nurse practitioner said: “Sometimes we really should be using the Language Line, but we do rely on family members, which is not the best practice. It gets the job done, but I don’t know what's being translated into” (#06).

The majority of providers stated that their clinics tried to overcome language barriers by providing in-person interpreter services for refugee women. In some cases, interpreters also assumed the job of being patient navigators for refugee women. A registered nurse said: “We did have a consistent translator who … would speak Pashto … She would help all these women navigate the system” (#10). Several providers discussed the positive impact of such services. A physician said: “For the Iraqi refugees, Syrian refugees, we have [Arabic-speaking] staff. And then we have staff from Afghanistan for the Afghani refugees. We have a couple of staff who speak Swahili … They’re hired on purpose to keep it a multilingual place, so that the patients feel comfortable” (#15). Another physician discussed: “Our Burmese navigator is here three days a week. So [the patients] want to come when the navigator is here … [The patients and the navigator] have a very strong relationship. That's great because it actually helps them with follow-ups and just with all around care” (#07).

Although providers recognized the benefits in providing in-person interpretation, it was not always feasible. A registered nurse explained: “At our clinic alone, patients speak approximately 40 different languages … there are a lot more challenges trying to deal with the broad range of diversity … It's both a good thing and a challenge at the same time” (#17). A nurse practitioner said: “[For patients that speak Arabic], occasionally we will have an interpreter on site, but not always. Some of the dialects of the African nations and the Bhutanese, Burmese can be kind of tough to find” (#05).

#### Limited capability to provide sexual and reproductive health services to refugee women

Most participants indicated their clinics were able to provide certain essential SRH services to refugee women, such as mammograms, Pap smears, sexually transmitted disease testing, or contraceptives. Clinics also frequently offered free or low-cost services or had sliding scale fees to assist with cost-related barriers for refugee women with low income. A physician said: “We don’t want money to be a barrier … We had a patient that just paid $5 because that's all they can pay” (#13).

A few clinics, however, faced policy constraints that limited who they could see. Therefore, even if women were able to access the clinic, they may not be able to utilize SRH services. A nurse practitioner explained: “We will only see people without insurance” (#02). Other clinic-level capacity barriers included a lack of human resources (e.g., a lack of specialists in obstetrics/gynecology) or providers’ time constraints. Some clinics operated primarily with volunteer providers, which limited the availability of appointments for refugee women. A nurse practitioner explained: “It would be great to have the clinic open even more hours. Right now it's somewhat limited … For the longest time, it was Sundays only and then it became Sundays and Fridays” (#01). A physician said: “[Patients] have to expect to be [waiting at the clinic] for probably about like three or four hours before you’re seen” (#08).

In addition, the majority of providers mentioned the lack of funding for, and consequently, the inadequate offering of SRH services at the clinics serving refugee women. The range of SRH services limited what refugee women can utilize. A physician stated: “It's just such a resource poor clinic … We don’t have ultrasound, we don’t have the ability to do prenatal care. We can’t do a lot of free birth control. Our microscope is crappy and you can’t see anything through it. There are a lot of things that could use improvement” (#08). Another physician remarked: “We can’t provide HIV testing and syphilis testing … that's one thing that we would really like to do. Being able to provide HPV vaccine [is] something we’d like to do” (#12).

#### Challenges with current referral system for SRH services

Most providers discussed referring female refugee patients to other settings (e.g., public hospitals, other FQHCs, or non-profit organizations) for services that were not provided at their particular clinics (e.g., prenatal care, HPV vaccination, and SRH diagnostic tests). Several providers remarked on the challenges with this type of “fragmented” care. A nurse practitioner brought up restrictions on where patients could be referred to based on their catchment areas: “If the person doesn’t live in DeKalb or Fulton County, we can’t refer them to Grady [Memorial Hospital]” (#02). A physician discussed other logistical difficulties: “If [patients] need more than just an annual exam or a basic treatment, then they have to go to Grady [Memorial Hospital] … talk to financial, get a Grady card, get scheduled and there's a waiting period involved” (#08). Moreover, a few providers reported poor inter-clinic communication of medical records and test results.

Several providers indicated barriers that may impede refugee women's follow-through on referrals. A physician said: “What we basically do is give [female refugee patients] information and just hope that they end up going there. But they don’t have the transportation, they don’t speak the language” (#08). A registered nurse stated: “Just how complex the structure of doing a referral is, patients having … to pick up on a number they might not know … Our patients, because they’re using pay-per-use cell phones, their phone numbers are changing every time they’re in clinic and so it makes doing referrals just very hard” (#17).

Patient navigators or case managers were helpful for ascertaining follow-through on referrals. A physician said: “If [the patients are] Burmese, Nepali, Congolese or Spanish speaking, then we have a navigator for those groups. The navigator will help make the appointment, follow through with the family and try to facilitate them going, even try to help with transport … Before we had navigators, we had a lower compliance and lower ability to refer. With the navigators … it's improved” (#07).

## Discussion

Our study documented metropolitan Atlanta-based healthcare providers’ perceptions of healthcare system-related influences on refugee women's access and utilization of SRH services. Findings highlighted the importance of clinic-level provision of transportation and interpretation services. At the same time, several providers noted a tension between women's preference for using friends or family members as informal interpreters and providers' desire to use more formal services, such as the Language Line. In addition, the majority of providers reported a lack of clinic-level capacity or resources to provide high-quality, comprehensive SRH care for refugee women. Several providers brought up challenges with using a referral system for refugee women to get additional SRH care in other healthcare settings. Patient navigators were described as a key facilitator that helped refugee women navigate the healthcare system and increase their SRH services access and utilization.

Our findings echo those of other studies with different populations in the U.S. that have recognized transportation difficulties as a central barrier to healthcare utilization (77, 78) in general and SRH services utilization in particular (79–82). In our study, only one clinic was able to offer transportation aid to refugee women. To ameliorate SRH disparities, it is imperative that clinics providing SRH care to refugee women can receive adequate funding and support to operate similar assistance programs (83, 84). In addition to traditional non-emergency medical transportation, initiatives such as rideshare (e.g., Uber or Lyft) can be considered (85). For example, one Boston-based pilot program found that coordinating rideshares for refugee and asylum seeking women is an effective and cost-efficient strategy to increase health services access (86). In case women need to be referred from one setting to another (as commonly described by providers in our study), it is possible that transportation services can increase refugee women's follow-through on referrals.

Besides the lack of transportation programs, providers in our study reported limitations in delivering high-quality, comprehensive SRH care for refugee women due to insurance policy, unavailability of appointments, or a lack of funding for several services. The few studies assessing system-level resources among organizations in the U.S. that serve refugees have primarily focused on non-medical organizations (e.g., local nonprofit organizations, voluntary or resettlement agencies) (87, 88). Our study adds to the literature by underscoring the current constraints of clinics in the U.S. that directly provided healthcare to refugee women. Several clinics in our study depended heavily on volunteers and limited grant funding for SRH services. Such operation is not financially sustainable because the capacity or availability of volunteers and the amount of funding can vary at any given time. More long-term, financially sustainable care models for refugee health should be explored in future research (89). Given the identified barriers to follow-through on referrals (e.g., restrictions based on catchment areas, logistical difficulties), additional research is needed to explore approaches to help refugee women overcome these barriers.

To our knowledge, this study is one of the first to explore perspectives of providers in Georgia or the Southeastern U.S. on refugee women's access and utilization of SRH services. We also note that providers in our study also served refugees from diverse backgrounds instead of from one country or ethnic group, which sets our research apart from past studies (31) and enhances the external validity of our findings. Much of existing U.S.-based research on refugee women's SRH access and utilization has focused on the coasts (90) or the Midwest (14), where healthcare resources and policy differ considerably from the setting of our study. Research with the context of Georgia is highly needed given the unique nature of the state with a large population of resettled refugees from diverse origins (54–58) but also poor healthcare access (e.g., third worst uninsured rate in the U.S.) (62) and restrictive healthcare policy (e.g., lack of full Medicaid expansion, restrictive abortion laws) (63, 91). While the scope of our study does not permit us to compare across settings (e.g., states), future research can consider how different state and local policy shape refugee women's experiences accessing and utilizing SRH care. For example, research can investigate whether state or local environments directly impact the funding and resources that clinics receive to provide SRH care for refugee women.

The importance of providing interpretation services during healthcare encounters has been noted in previous studies with providers serving refugee women in the U.S. (45, 46). We found a tension between refugee women's preferred usage of informal interpretation assistance and healthcare providers' desire for more formal interpretation services. This discordance points to a need to assess the acceptability of healthcare provision in clinical settings from multiple perspectives, especially with populations with diverse cultural backgrounds. While not specific to the U.S. context and SRH care delivery, a few previous studies in international settings with other health issues have noted similar concerns that providers had with refugee patients' family members or friends serving as interpreters (e.g., safety, confidentiality, or translation accuracy) (92–94). A study with Somali Bantu women in Connecticut also discusses several issues with using the Language Line for translating reproductive healthcare encounters (90), For example, the translators identified Af Maxaa as the “Somali” language as it is spoken by the majority of Somali immigrants. Somali Bantu women, however, use Af Maay, a language that share some similarities in the written form to Af Maxaa but is distinct enough in the spoken form such that the two languages are mutually unintelligible (90). In addition, sometimes the translation is done by a male translator which made women hesitant to discuss SRH (90).

Based on their experiences, providers in our study believed patient navigators can effectively address language-related challenges and that refugee women were comfortable with having patient navigators be their translators in clinical encounters. Moreover, patient navigators were critical in ensuring follow-through on referrals (e.g., helping women overcome issues in the process of going from a FQHC to a public hospital for additional SRH care). In the U.S., the role of patient navigators was historically created in the context of cancer screening, diagnosis, and care. Patient navigators are an asset to the communities they work in and help patients and caregivers comply with evidence-based guidelines for cancer prevention and early detection (95).

Patient navigators facilitate access for underserved populations to healthcare systems and connect them to resources most appropriate for their needs (95). Specific to refugee populations in the U.S., the use of patient navigators or community health workers has been shown to reduce barriers to healthcare access and improved healthcare in the context of cancer screenings and primary care services (96–99). Findings from our study contribute to the literature by highlighting the fact that patient navigators can be helpful outside of the context of cancer screenings and primary care services – specifically, they can be a key facilitator in assisting refugee women with navigating the healthcare system for SRH. As Davidson and colleagues have found in their systematic review, one of the critical barriers refugee women in high-income host countries face is related to navigating and understanding the health system and making appointments for SRH care (31).

These findings call for additional research using an implementation science perspective and exploring organizational and external factors that can help or hinder the adoption of patient navigators for refugee women's SRH care. Existing implementation studies on patient navigation, while not focusing specifically on SRH, have identified several critical factors that should be considered such as planning to ensure alignment with organizational need, integration of the navigator into clinical processes, appropriate caseload, multidisciplinary engagement, funding, and in-kind resources (100, 101). Although the use of patient navigators holds potential for increasing refugee women's SRH care access and utilization, several issues may remain over the large-scale implementation of this model.

For example, given the incredible diversity of refugee patients in Atlanta, it may be challenging to have navigators for each different language or dialect that patients spoke. In addition, while we did not specifically inquire about costs in our study, many patient navigation programs currently lack stable funding and rely on inconsistent sources of payment (102). Given the promise of patient navigation in improving outcomes and reducing disparities, future work can explore scalable implementation strategies and cost-effectiveness of navigation programs for refugee populations (102, 103). Moreover, models using multisectoral partnerships to consolidate resources and coordinate navigation services between different healthcare entities and community-based or nonprofit organizations (e.g., between hospitals, resettlement agencies, and primary care practices) have been shown to be financially sustainable (89).

### Strengths and limitations

The strengths of our study include the use of multilevel theoretical frameworks and rigorous qualitative research procedures. We sampled providers with different clinical roles and from different clinical settings; providers in our study also served refugees from diverse backgrounds as opposed to one particular population. Nevertheless, given the heterogeneity of experiences, our study may face limitations in terms of the transferability of results to different settings and contexts (104). Specifically, the context of Georgia (high concentration of refugees, poor healthcare access, and restrictive healthcare policy) may limit the transferability of our results. While the perspectives of providers are critical to understanding organizational contexts, in this manuscript we could not include refugee women's insights on other important factors that have been noted to influence SRH access and utilization (e.g., sociocultural factors, trust, and the refugee experience) (31). Davidson and colleagues' systematic review also noted the significant influence of providers' characteristics and past cultural competence training (31), which will be the focus of additional manuscripts from our study. We acknowledge that the positioning of the researchers in relation to the community of study affected how the research question was formed, who was invited to participate, and the way in which knowledge was constructed and disseminated (105). While we determined an *a priori* sample size based on previous relevant studies and recommendations for saturation in qualitative research, it is possible that a larger sample size would have enhanced data adequacy (106).

## Conclusion

Our study explores healthcare providers’ perspectives of healthcare system-related factors influencing refugee women's access and utilization of SRH services. Findings underscore current constraints of clinics that provided SRH care to refugee women. The provision of transportation services and acceptable interpretation services holds potential for increasing refugee women's SRH care access and utilization. Implementing patient navigation can reduce language-related challenges for refugee women and help women overcome issues to navigate the healthcare system for SRH.

## Data Availability

The data that support the findings of this study are available upon reasonable request and with the approval of Emory University Institutional Review Board. The data are not publicly available as it contains information that could compromise the privacy of research participants. Please contact Dr. Cam Escoffery (cescoff@emory.edu) with any request for data access.
